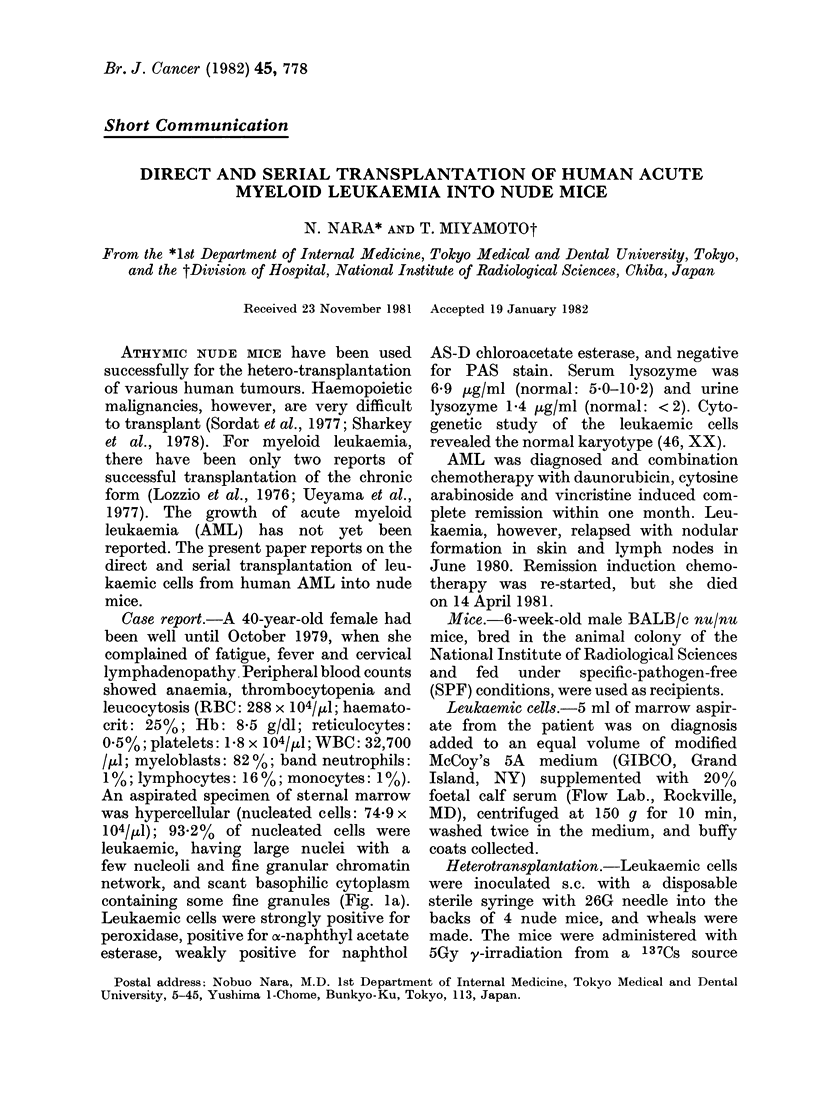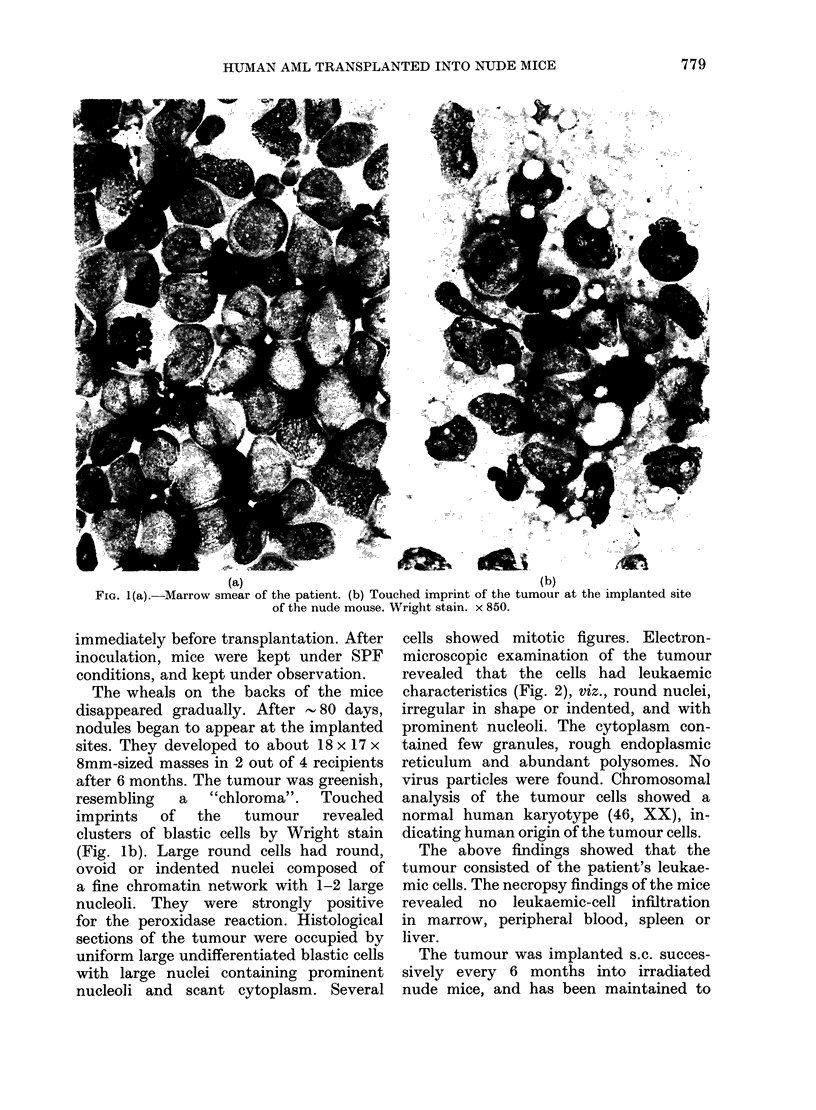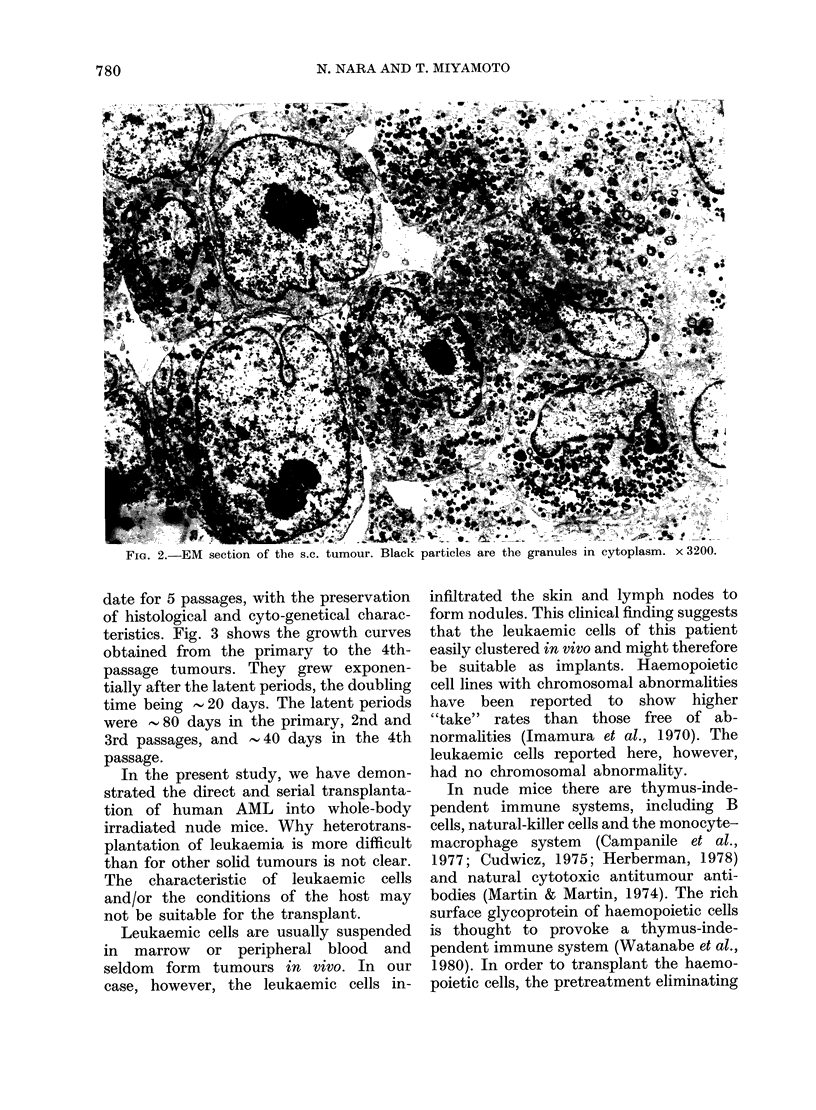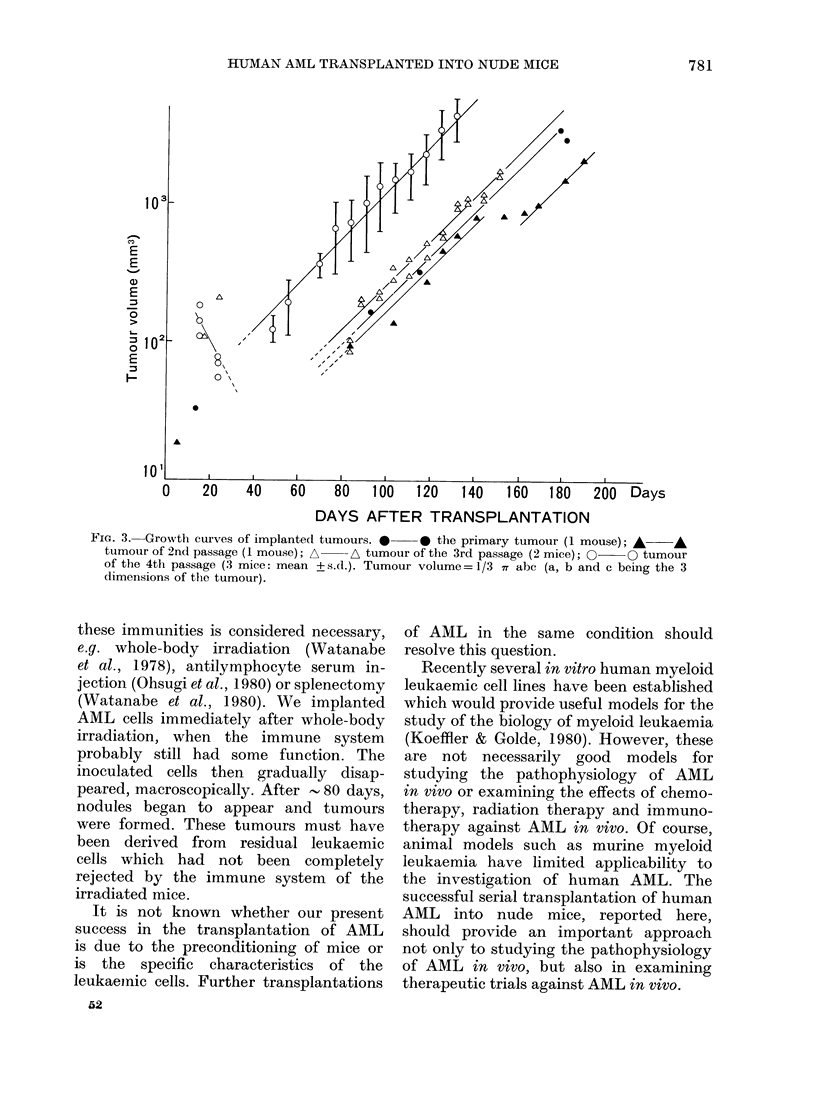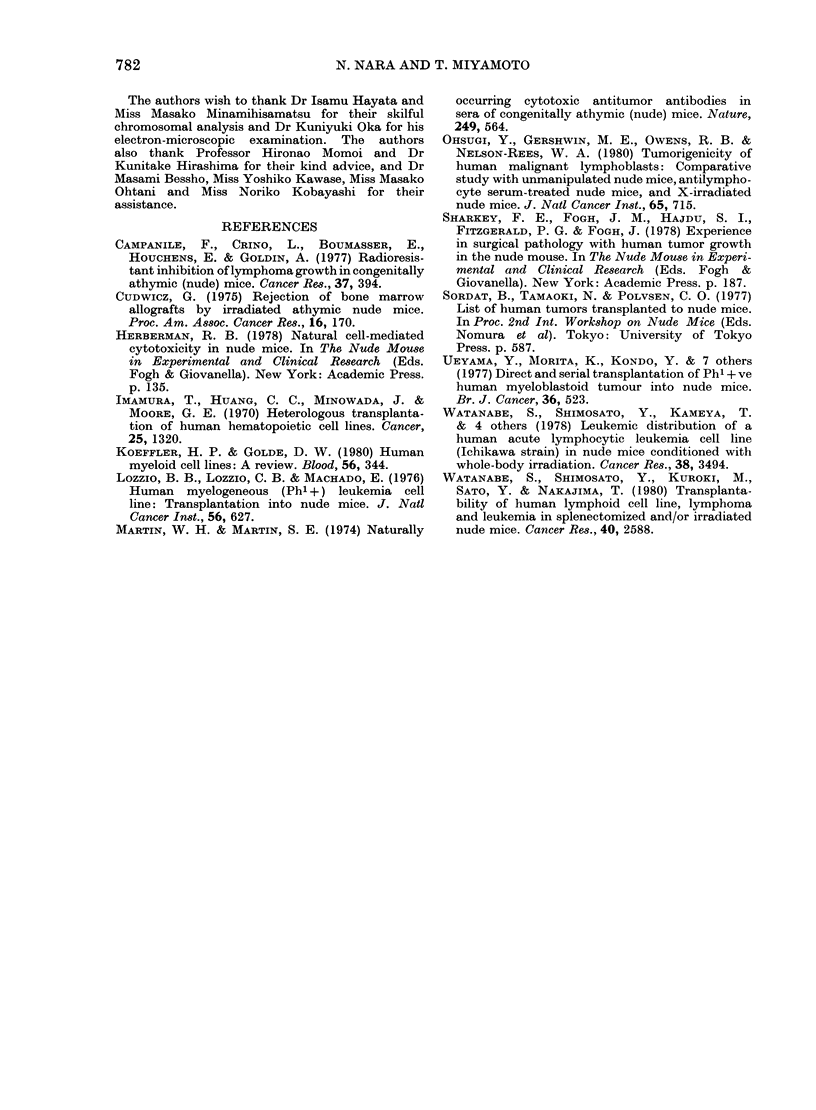# Direct and serial transplantation of human acute myeloid leukaemia into nude mice.

**DOI:** 10.1038/bjc.1982.120

**Published:** 1982-05

**Authors:** N. Nara, T. Miyamoto

## Abstract

**Images:**


*Br. J. Cancer* (1982) **45**, 778

***Short Communication***

### DIRECT AND SERIAL TRANSPLANTATION OF HUMAN ACUTE MYELOID LEUKAEMIA INTO NUDE MICE

N. NARA* AND T. MIYAMOTO†

*From the *1st Department of Internal Medicine, Tokyo Medical and Dental University, Tokyo, and the †Division of Hospital, National Institute of Radiological Sciences, Chiba, Japan*

Received 23 November 1981 Accepted 19 January 1982

ATHYMIC NUDE MICE have been used successfully for the hetero-transplantation of various human tumours. Haemopoietic malignancies, however, are very difficult to transplant (Sordat *et al.*, 1977; Sharkey *et al.*, 1978). For myeloid leukaemia, there have been only two reports of successful transplantation of the chronic form (Lozzio *et al.*, 1976; Ueyama *et al.*, 1977). The growth of acute myeloid leukaemia (AML) has not yet been reported. The present paper reports on the direct and serial transplantation of leukaemic cells from human AML into nude mice.

*Case report.*—A 40-year-old female had been well until October 1979, when she complained of fatigue, fever and cervical lymphadenopathy. Peripheral blood counts showed anaemia, thrombocytopenia and leucocytosis (RBC: $288 \times 10^4/\mu l$; haematocrit: 25%; Hb: 8·5 g/dl; reticulocytes: 0·5%; platelets: $1{\cdot}8 \times 10^4/\mu l$; WBC: 32,700 /$\mu$l; myeloblasts: 82%; band neutrophils: 1%; lymphocytes: 16%; monocytes: 1%). An aspirated specimen of sternal marrow was hypercellular (nucleated cells: $74{\cdot}9 \times 10^4/\mu l$); 93·2% of nucleated cells were leukaemic, having large nuclei with a few nucleoli and fine granular chromatin network, and scant basophilic cytoplasm containing some fine granules (Fig. 1a). Leukaemic cells were strongly positive for peroxidase, positive for $\alpha$-naphthyl acetate esterase, weakly positive for naphthol AS-D chloroacetate esterase, and negative for PAS stain. Serum lysozyme was 6·9 $\mu$g/ml (normal: 5·0–10·2) and urine lysozyme 1·4 $\mu$g/ml (normal: $< 2$). Cytogenetic study of the leukaemic cells revealed the normal karyotype (46, XX).

AML was diagnosed and combination chemotherapy with daunorubicin, cytosine arabinoside and vincristine induced complete remission within one month. Leukaemia, however, relapsed with nodular formation in skin and lymph nodes in June 1980. Remission induction chemotherapy was re-started, but she died on 14 April 1981.

*Mice.*—6-week-old male BALB/c *nu/nu* mice, bred in the animal colony of the National Institute of Radiological Sciences and fed under specific-pathogen-free (SPF) conditions, were used as recipients.

*Leukaemic cells.*—5 ml of marrow aspirate from the patient was on diagnosis added to an equal volume of modified McCoy's 5A medium (GIBCO, Grand Island, NY) supplemented with 20% foetal calf serum (Flow Lab., Rockville, MD), centrifuged at 150 *g* for 10 min, washed twice in the medium, and buffy coats collected.

*Heterotransplantation.*—Leukaemic cells were inoculated s.c. with a disposable sterile syringe with 26G needle into the backs of 4 nude mice, and wheals were made. The mice were administered with 5Gy $\gamma$-irradiation from a $^{137}Cs$ source

Postal address: Nobuo Nara, M.D. 1st Department of Internal Medicine, Tokyo Medical and Dental University, 5–45, Yushima 1-Chome, Bunkyo-Ku, Tokyo, 113, Japan.

FIG. 1(a).—Marrow smear of the patient. (b) Touched imprint of the tumour at the implanted site of the nude mouse. Wright stain. × 850.

immediately before transplantation. After inoculation, mice were kept under SPF conditions, and kept under observation.

The wheals on the backs of the mice disappeared gradually. After ~80 days, nodules began to appear at the implanted sites. They developed to about 18 × 17 × 8mm-sized masses in 2 out of 4 recipients after 6 months. The tumour was greenish, resembling a "chloroma". Touched imprints of the tumour revealed clusters of blastic cells by Wright stain (Fig. 1b). Large round cells had round, ovoid or indented nuclei composed of a fine chromatin network with 1–2 large nucleoli. They were strongly positive for the peroxidase reaction. Histological sections of the tumour were occupied by uniform large undifferentiated blastic cells with large nuclei containing prominent nucleoli and scant cytoplasm. Several cells showed mitotic figures. Electron-microscopic examination of the tumour revealed that the cells had leukaemic characteristics (Fig. 2), *viz.*, round nuclei, irregular in shape or indented, and with prominent nucleoli. The cytoplasm contained few granules, rough endoplasmic reticulum and abundant polysomes. No virus particles were found. Chromosomal analysis of the tumour cells showed a normal human karyotype (46, XX), indicating human origin of the tumour cells.

The above findings showed that the tumour consisted of the patient's leukaemic cells. The necropsy findings of the mice revealed no leukaemic-cell infiltration in marrow, peripheral blood, spleen or liver.

The tumour was implanted s.c. successively every 6 months into irradiated nude mice, and has been maintained to

FIG. 2.—EM section of the s.c. tumour. Black particles are the granules in cytoplasm. ×3200.

date for 5 passages, with the preservation of histological and cyto-genetical characteristics. Fig. 3 shows the growth curves obtained from the primary to the 4th-passage tumours. They grew exponentially after the latent periods, the doubling time being ~20 days. The latent periods were ~80 days in the primary, 2nd and 3rd passages, and ~40 days in the 4th passage.

In the present study, we have demonstrated the direct and serial transplantation of human AML into whole-body irradiated nude mice. Why heterotransplantation of leukaemia is more difficult than for other solid tumours is not clear. The characteristic of leukaemic cells and/or the conditions of the host may not be suitable for the transplant.

Leukaemic cells are usually suspended in marrow or peripheral blood and seldom form tumours *in vivo*. In our case, however, the leukaemic cells infiltrated the skin and lymph nodes to form nodules. This clinical finding suggests that the leukaemic cells of this patient easily clustered *in vivo* and might therefore be suitable as implants. Haemopoietic cell lines with chromosomal abnormalities have been reported to show higher "take" rates than those free of abnormalities (Imamura *et al.*, 1970). The leukaemic cells reported here, however, had no chromosomal abnormality.

In nude mice there are thymus-independent immune systems, including B cells, natural-killer cells and the monocyte–macrophage system (Campanile *et al.*, 1977; Cudwicz, 1975; Herberman, 1978) and natural cytotoxic antitumour antibodies (Martin & Martin, 1974). The rich surface glycoprotein of haemopoietic cells is thought to provoke a thymus-independent immune system (Watanabe *et al.*, 1980). In order to transplant the haemopoietic cells, the pretreatment eliminating

FIG. 3.—Growth curves of implanted tumours. ●——● the primary tumour (1 mouse); ▲——▲ tumour of 2nd passage (1 mouse); △——△ tumour of the 3rd passage (2 mice); ○——○ tumour of the 4th passage (3 mice: mean ±s.d.). Tumour volume = 1/3 π abc (a, b and c being the 3 dimensions of the tumour).

these immunities is considered necessary, *e.g.* whole-body irradiation (Watanabe *et al.*, 1978), antilymphocyte serum injection (Ohsugi *et al.*, 1980) or splenectomy (Watanabe *et al.*, 1980). We implanted AML cells immediately after whole-body irradiation, when the immune system probably still had some function. The inoculated cells then gradually disappeared, macroscopically. After ~80 days, nodules began to appear and tumours were formed. These tumours must have been derived from residual leukaemic cells which had not been completely rejected by the immune system of the irradiated mice.

It is not known whether our present success in the transplantation of AML is due to the preconditioning of mice or is the specific characteristics of the leukaemic cells. Further transplantations of AML in the same condition should resolve this question.

Recently several *in vitro* human myeloid leukaemic cell lines have been established which would provide useful models for the study of the biology of myeloid leukaemia (Koeffler & Golde, 1980). However, these are not necessarily good models for studying the pathophysiology of AML *in vivo* or examining the effects of chemotherapy, radiation therapy and immunotherapy against AML *in vivo*. Of course, animal models such as murine myeloid leukaemia have limited applicability to the investigation of human AML. The successful serial transplantation of human AML into nude mice, reported here, should provide an important approach not only to studying the pathophysiology of AML *in vivo*, but also in examining therapeutic trials against AML *in vivo*.

The authors wish to thank Dr Isamu Hayata and Miss Masako Minamihisamatsu for their skilful chromosomal analysis and Dr Kuniyuki Oka for his electron-microscopic examination. The authors also thank Professor Hironao Momoi and Dr Kunitake Hirashima for their kind advice, and Dr Masami Bessho, Miss Yoshiko Kawase, Miss Masako Ohtani and Miss Noriko Kobayashi for their assistance.

### REFERENCES

CAMPANILE, F., CRINO, L., BOUMASSER, E., HOUCHENS, E. & GOLDIN, A. (1977) Radioresistant inhibition of lymphoma growth in congenitally athymic (nude) mice. *Cancer Res.*, **37**, 394.

CUDWICZ, G. (1975) Rejection of bone marrow allografts by irradiated athymic nude mice. *Proc. Am. Assoc. Cancer Res.*, **16**, 170.

HERBERMAN, R. B. (1978) Natural cell-mediated cytotoxicity in nude mice. In *The Nude Mouse in Experimental and Clinical Research* (Eds. Fogh & Giovanella). New York: Academic Press. p. 135.

IMAMURA, T., HUANG, C. C., MINOWADA, J. & MOORE, G. E. (1970) Heterologous transplantation of human hematopoietic cell lines. *Cancer*, **25**, 1320.

KOEFFLER, H. P. & GOLDE, D. W. (1980) Human myeloid cell lines: A review. *Blood*, **56**, 344.

LOZZIO, B. B., LOZZIO, C. B. & MACHADO, E. (1976) Human myelogeneous ($Ph^1+$) leukemia cell line: Transplantation into nude mice. *J. Natl Cancer Inst.*, **56**, 627.

MARTIN, W. H. & MARTIN, S. E. (1974) Naturally occurring cytotoxic antitumor antibodies in sera of congenitally athymic (nude) mice. *Nature*, **249**, 564.

OHSUGI, Y., GERSHWIN, M. E., OWENS, R. B. & NELSON-REES, W. A. (1980) Tumorigenicity of human malignant lymphoblasts: Comparative study with unmanipulated nude mice, antilymphocyte serum-treated nude mice, and X-irradiated nude mice. *J. Natl Cancer Inst.*, **65**, 715.

SHARKEY, F. E., FOGH, J. M., HAJDU, S. I., FITZGERALD, P. G. & FOGH, J. (1978) Experience in surgical pathology with human tumor growth in the nude mouse. In *The Nude Mouse in Experimental and Clinical Research* (Eds. Fogh & Giovanella). New York: Academic Press. p. 187.

SORDAT, B., TAMAOKI, N. & POLVSEN, C. O. (1977) List of human tumors transplanted to nude mice. In *Proc. 2nd Int. Workshop on Nude Mice* (Eds. Nomura *et al*). Tokyo: University of Tokyo Press. p. 587.

UEYAMA, Y., MORITA, K., KONDO, Y. & 7 others (1977) Direct and serial transplantation of $Ph^1$ + ve human myeloblastoid tumour into nude mice. *Br. J. Cancer*, **36**, 523.

WATANABE, S., SHIMOSATO, Y., KAMEYA, T. & 4 others (1978) Leukemic distribution of a human acute lymphocytic leukemia cell line (Ichikawa strain) in nude mice conditioned with whole-body irradiation. *Cancer Res.*, **38**, 3494.

WATANABE, S., SHIMOSATO, Y., KUROKI, M., SATO, Y. & NAKAJIMA, T. (1980) Transplantability of human lymphoid cell line, lymphoma and leukemia in splenectomized and/or irradiated nude mice. *Cancer Res.*, **40**, 2588.